# SMoRe ParS: A novel methodology for bridging modeling modalities and experimental data applied to 3D vascular tumor growth

**DOI:** 10.3389/fmolb.2022.1056461

**Published:** 2022-12-23

**Authors:** Harsh Vardhan Jain, Kerri-Ann Norton, Bernardo Bianco Prado, Trachette L. Jackson

**Affiliations:** ^1^ Department of Mathematics and Statistics, University of Minnesota Duluth, Duluth, MN, United States; ^2^ Reem and Kayden Center for Science and Computation, Computational Biology Laboratory, Computer Science Program, Bard College, Annandale-on-Hudson, NY, United States; ^3^ Department of Mathematics, University of Michigan, Ann Arbor, MI, United States

**Keywords:** agent-based model, parameter identifiability, surrogate model, uncertainty quantification, vascular tumor growth

## Abstract

Multiscale systems biology is having an increasingly powerful impact on our understanding of the interconnected molecular, cellular, and microenvironmental drivers of tumor growth and the effects of novel drugs and drug combinations for cancer therapy. Agent-based models (ABMs) that treat cells as autonomous decision-makers, each with their own intrinsic characteristics, are a natural platform for capturing intratumoral heterogeneity. Agent-based models are also useful for integrating the multiple time and spatial scales associated with vascular tumor growth and response to treatment. Despite all their benefits, the computational costs of solving agent-based models escalate and become prohibitive when simulating millions of cells, making parameter exploration and model parameterization from experimental data very challenging. Moreover, such data are typically limited, coarse-grained and may lack any spatial resolution, compounding these challenges. We address these issues by developing a first-of-its-kind method that leverages explicitly formulated surrogate models (SMs) to bridge the current computational divide between agent-based models and experimental data. In our approach, Surrogate Modeling for Reconstructing Parameter Surfaces (SMoRe ParS), we quantify the uncertainty in the relationship between agent-based model inputs and surrogate model parameters, and between surrogate model parameters and experimental data. In this way, surrogate model parameters serve as intermediaries between agent-based model input and data, making it possible to use them for calibration and uncertainty quantification of agent-based model parameters that map directly onto an experimental data set. We illustrate the functionality and novelty of Surrogate Modeling for Reconstructing Parameter Surfaces by applying it to an agent-based model of 3D vascular tumor growth, and experimental data in the form of tumor volume time-courses. Our method is broadly applicable to situations where preserving underlying mechanistic information is of interest, and where computational complexity and sparse, noisy calibration data hinder model parameterization.

## 1 Introduction

Validated mathematical models of tumor growth mediated by complex microenvironmental interactions and signals are increasingly being recognized as an invaluable aid for elucidating mechanisms underpinning experimental and clinical observations (Byrne, 2010; Franssen et al., 2019; Butner et al., 2020; Butner et al., 2021). These models often use continuum ordinary or partial differential equations (ODEs/PDEs) to predict cancer cell number (or densities) in time and/or space. Continuum approaches are a common choice because they allow for rapid simulation and open the door to advanced analyses (global sensitivity, structural and practical identifiability, bifurcations, etc.) that reveal key parameter relationships. They also enable the use of time-course experimental data for parameter estimation and model validation (Brouwer et al., 2017; Eisenberg and Jain, 2017).

An alternative approach is a discretized method that models cells as autonomous, decision making “agents,” each with their own set of properties and behaviors. These agent-based models (ABMs) have become a valuable tool in translational systems oncology, which has goals of predicting the effects of novel drugs and drug combinations on difficult-to-treat tumors (Altrock et al., 2015; Wang et al., 2015; Bergman et al., 2022). ABMs provide a logical structure for capturing the multiple time and spatial scales associated with cancer growth and progression because they allow for the characterization of tumor heterogeneity at an individual cell level that better reflects the complexity seen *in vivo* (Bergman et al., 2022). One major advantage of ABMs over traditional continuum ODE/PDE models is that they can generate realistic 3-dimensional virtual tumors that current state-of-art imaging technologies cannot infer from patient scans [for a discussion on limitations of imaging in cancer, see for instance (Bogdanovic et al., 2021; Ding et al., 2021; Martinez-Heras et al., 2021)]. However, to make useful, reliable quantitative predictions, ABMs need to relate to real-world data through model parameterization and calibration (Byrne, 2010; Eisenberg and Jain, 2017). Unfortunately, a significant limitation of these models is that they can be computationally expensive, especially as the number of agents (cells) expands. Computational times and memory requirements can become prohibitive when simulating upwards of 10^6^–10^7^ agents (Ghaffarizadeh et al., 2018). This is in direct opposition to the fact that just one cubic centimeter of tissue will contain 10^8^–10^9^ cells and many *in vivo* experiments begin with 10^4^–10^6^ cells (Del Monte, 2009). These computational costs are exacerbated when ABMs include molecular level details of cell signaling or targeted therapeutics Ghaffarizadeh et al. (2018). The inherent stochasticity and heavy computational requirements of an ABM are significant obstacles for data-driven parameterization and for conducting rigorous parameter space exploration and sensitivity analyses (Norton and Popel, 2016; Zhang et al., 2020; Broniec et al., 2021). Moreover, experimental data is typically limited, coarse-grained and may lack any spatial resolution, resulting in issues of parameter identifiability (Eisenberg and Jain, 2017).

There is hence a need for developing new theoretical and computational frameworks that can bridge this gap between ABM parameters and real-world data. Estimating ABM parameters from noisy experimental data is particularly challenging because ABM behavior emerges from interactions among many individuals and the computational expense scales with the number of parameters (Broniec et al., 2021). One approach for exploring ABMs is to run extensive Monte Carlo simulations, but this is infeasible for complex models (Nardini et al., 2021). Bayesian methods are not ideal because they rely on prior knowledge about the probability distributions of the components being modeled, which is rarely available (Broniec et al., 2021). Some researchers have used genetic algorithms (GA) together with agent-based models for parameter space exploration and parameter estimation (Calvez and Hutzler, 2005; Lee et al., 2015); however, GAs require a very large number of iterations to converge, thus exacerbating computational expense issues (Broniec et al., 2021). Yet another approach entails the derivation of coarse-grained ODE/PDEs (mean-field models) to predict average outputs of the ABM. However, such mean-field models typically fail to accurately describe ABM dynamics in certain parameter regimes (Klank et al., 2018; Nardini et al., 2021).

To address some of these challenges, we develop an approach that uses an explicitly formulated surrogate model (SM) that will bridge ABM simulations and experimental data. Surrogate models (also called metamodels or response surfaces) are computationally cheaper models designed to approximate the dominant features of a complex model, here, the ABM (Blanning, 1975; Regis and Shoemaker, 2005; O’Hagan, 2006; Asher et al., 2015). They have been used extensively in engineering applications (see (Palar et al., 2019) for a review) and weather forecasting [see (Vlahogianni, 2015; Schultz et al., 2021) for recent reviews]. Specifically, we employ model selection to infer an SM directly from both ABM output and experimental data so that we accurately capture aggregate ABM dynamics. In our approach, Surrogate Modeling for Reconstructing Parameter Surfaces (SMoRe ParS), we quantify the relationship between parameter values across the two types of models (ABM and SM) and between SM parameters and experimental data. Thus, SM parameters act as interlocutors between ABM inputs and data that can be used for calibration and uncertainty quantification of ABM parameters.

Indeed, parameterizing ABMs with SMs that use machine learning algorithms, where the SM does not have a closed form, is becoming increasingly popular. Using examples from finance, (Lamperti et al., 2018; Zhang et al., 2020) describe a surrogate modeling method for ABM calibration that combines supervised machine-learning and iterative sampling. These methods can learn a surrogate model as the approximation of the original system with a relatively small number of training points by using an iterative sampling algorithm that intelligently searches the response surface. In (Perumal and van Zyl, 2020), different sampling methods and SMs derived from machine learning algorithms are integrated with a temporal ABM that describes infectious disease epidemiology to test how these strategies affect parameter space exploration. They show that surrogate assisted methods perform better than standard sampling methods in that they better identify the most likely parameter vector by matching the synthetic data distribution it generates with a real data distribution.

Our method differs from the approaches mentioned above in several ways. Two major differentiators of our approach are: 1) The SM equations are explicitly formulated, this formulation being informed by the experimental data; and 2) SM parameters are distinctly mapped to both, the ABM the input parameters, as well as the calibration data set. In this way, our strategy enables the SM to be informed by both the ABM output, and the experimental data. We also infer ABM parameter regions that correspond to the data and propagate uncertainty *via* SM parameters to ABM parameters. Finally, by making such an explicit connection between ABM input, SM parameters and the data, we can account for inherent differences in dimensionality or physical units between ABM output and experimental data.

In the sections below we describe the details of our new methods for narrowing the current divide between computationally intense, difficult to analyze/parameterize computational modeling approaches and experimental data. We then demonstrate the usefulness and novelty of our approach by applying it to an ABM of vascular tumor growth and experimental data in the form of tumor volume time-courses.

## 2 Methods

### 2.1 Surrogate modeling for reconstructing parameter surfaces (SMoRe ParS)

**FIGURE 1:**
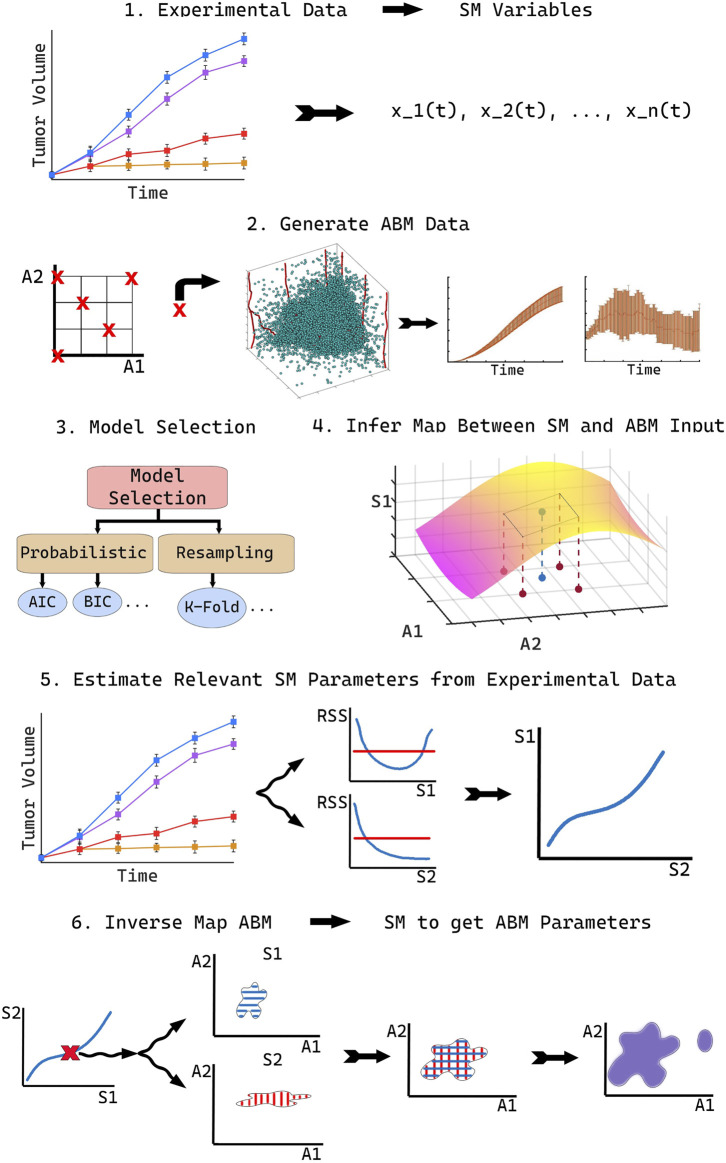
Schematic for implementing the SMoRe ParS method.

To accurately compare ABM output with real world data, we propose our novel methodology, SMoRe ParS. A schematic diagram of the full approach is provided in Figure 1. SMoRe ParS is a six-step strategy that users can implement as follows.

**Step 1: Use real-world data to inform SM formulation and variables**

First, determine the formulation of the SM from a real-world (experimental) data set. In particular, the goal is to determine both, the type of model to use (ODE, PDE, Boolean, etc.), and the variables needed for the model formation. For instance, time-course data would suggest a system of ODEs, whereas spatially resolved data might accommodate a PDE SM. Additionally, the quantities measured in the data set should inform the choice of SM variables. For instance, tumor volume measurements would suggest tumor cell numbers as a SM variable.

**Step 2: Generate ABM data**

In this step, identify a subset of ABM parameters of interest, say ${\vec{p}}_{\text{ABM}}$ , based on some predetermined criteria. For instance, in a model of chemotherapy, one might select parameters such as cancer cell proliferation rate and death rate, that is the input parameters that are directly relevant to the treatment of interest. Next, generate ABM output for a broad range of the chosen parameter values. Specifically, vary ABM parameters one at a time to sample along the boundary of the parameter space, and also select several parameter combinations at non-boundary points, to generate reference points in the interior. For each parameter combination, the ABM should be simulated multiple times to get meaningful average behavior. Finally, process the generated ABM output for inherent differences in dimensionality or physical units between ABM output and SM variables, if necessary. For instance, if the ABM output is a spatially resolved time-course of a growing tumor and a variable in the SM is total number of tumor cells as a function of time alone, then the number of tumor cells in the ABM simulations should be integrated over its spatial domain.

**Step 3: Perform SM model selection**

Select several potential models as SM candidates and test their ability to capture both the experimental data and the ABM output. Then perform model selection to arrive at a “most likely” SM. There are numerous model selection approaches to choose from when selecting the best model to move forward with, including probabilistic Information Criteria (Anderson and Burnham, 2004; Burnham and Anderson, 2004) or resampling methods (Efron, 1983; Shao, 1996). Others (Nardini et al., 2021) have proposed learning equations directly from data as a method to arrive at a consensus model.

**Step 4: Reconstruct SM parameter surfaces from ABM output**

Next, infer a quantitative relationship between each of the SM input parameters, ${\vec{p}}_{\text{SM}}=\left\langle p_{\text{SM},1},\ldots ,p_{\text{SM},i},\ldots ,p_{\text{SM},n}\right\rangle$ and selected ABM parameters, ${\vec{p}}_{\text{ABM}}$ . This is done by fitting SM parameters to ABM output generated in Step 2, for instance by performing maximum likelihood estimation (MLE) (Millar, 2011). A key advantage of our method is that any uncertainty in SM parameters is also quantified in this step. For example, if MLE is used to estimate SM parameter values, then the profile likelihood approach Eisenberg and Jain (2017) can be employed to quantify this uncertainty.

At this stage, for a given SM parameter *p*_SM,*i*_, estimates for its appropriate range of values (e.g., 95% confidence bounds) should be calculated at each of the sampled ABM parameter combinations. Assuming that *p*_SM,*i*_ and its confidence bounds (C.B._*i*_) are continuous but unknown functions of the ABM parameters ${\vec{p}}_{\text{ABM}}$ , reconstruct these functions—or hypersurfaces—as follows. The 95% confidence bound estimates found above correspond to discrete points on the upper and lower 95% confidence hypersurfaces (see Step 4 in Figure 1). Now, “fill in” the unknown upper and lower hypersurfaces, for instance, using polynomial or quadratic interpolation [see (Smith, 2013) for an overview of these methods]. That is, reconstruct parameter response surfaces that *p*_SM,*i*_ lies within. The completion of this step will result in an explicit (numerical) relationship between SM parameters and ABM parameters, that also preserves information on uncertainty in the SM parameters. That is:

$$p_{\text{SM},i}=f_{i}\left({\vec{p}}_{\text{ABM}}\right)\;\pm \;{\text{C.B.}}_{i}\left({\vec{p}}_{\text{ABM}}\right).$$ (1)

In the above hypersurface relationship, the function *f*_*i*_ is not explicitly determined, rather, it is numerically approximated.

**Step 5: Estimate SM parameters from real-world data**

In the next step, estimate values of SM input parameters ${\vec{p}}_{\text{SM}}$ from the real-world data, for instance by performing maximum likelihood estimation (MLE) as in the previous step. Ideally at this stage, practical identifiability of the SM model should be investigated to arrive at identifiable combinations of SM input parameters. Practical identifiability examines how real-world considerations, such as noise or sampling frequency, affect one’s ability to uniquely estimate model parameters from a given data set (Jacquez and Greif, 1985). This additional step of finding the practically identifiable combinations of SM parameters will help constrain the desired ABM parameter space that maps to real-world data in the next step.

**Step 6: Infer regions of ABM parameter space that correspond to real-world data**

In the final step of SMoRe ParS, overlay the ranges on data-derived SM parameters in the previous step on the inferred relationship between SM parameters and ABM parameters found in Step 4. This yields regions of ABM parameter space that correspond to experimental data. Specifically, for each data-informed choice of SM parameter vector ${\vec{p}}_{\text{SM}}$ , regions in ABM parameter-hyperspace are obtained *via* projection mapping for all its components, *p*_SM,*i*_. The intersection of these regions yields ABM parameter ranges that correspond to that specific choice of ${\vec{p}}_{\text{SM}}$ . Repeat this for several choices of ${\vec{p}}_{\text{SM}}$ —constrained by the practical identifiability information from Step 5—and take the union of the resulting ABM regions to arrive at the desired ranges in parameter values that match with the experimental data.

### 2.2 Proof of concept: Vascular tumor growth

In this section we work through the set up of a detailed proof of concept of our new method. To demonstrate the functionality and originality of our approach, we apply it to a 3D, multi-grid, on-lattice ABM of stem-cell driven vascular tumor growth.

#### 2.2.1 SM formulation and variables

We use experimental data from the breast cancer literature in the form of tumor volume time-courses [see Figure 5A in (Zhou et al., 2019)]. These data suggest that a single ODE tracking the number of tumor cells over time is an appropriate formulation for the SM.

#### 2.2.2 ABM formulation

**FIGURE 2:**
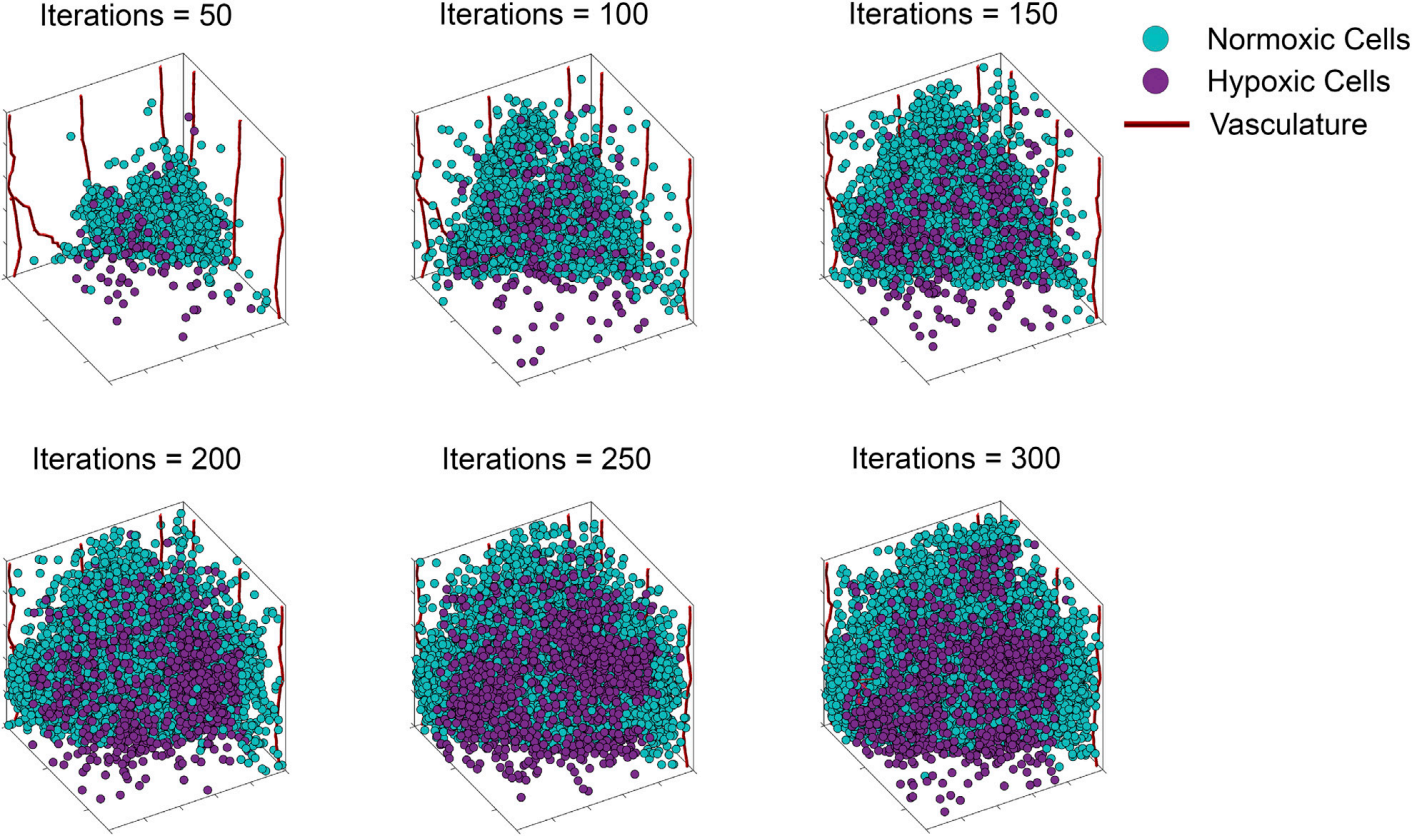
Storyboard showing a typical ABM simulation of vascular tumor growth, depicting the locations in space of normoxic (teal circles) and hypoxic (purple circles) tumor cells, along with vasculature (red curves).

The ABM of vascular tumor growth implemented here is a simplified form of previous models (Norton et al., 2018; Ventoso and Norton, 2020; Fischel et al., 2021). The simplified version consists of two modules: a tumor module and a vasculature module. Both modules are on-lattice, but they occupy different grids. The overall simulation domain is a cube of side 1 mm. As in (Norton et al., 2018), the tumor is initiated with cancer cells, progenitor and stem, placed in one corner of the grid. Cells cannot leave the boundary of the simulation domain. The vascular network at tumor initiation consists of mature vessels, each comprised of individual segments located along the grid boundaries closest to the initial tumor. This initial set up is visualized in Figure 2. The ABM simulates a tumor growing on the surface of healthy, vascularized tissue, which acts as an additional source of oxygen. The simulation is run for 300 iterations, each iteration corresponding to ∼6 h. For more information on model assumptions, setup and simulation methodology, we refer the reader to (Norton et al., 2017; Ventoso and Norton, 2020; Fischel et al., 2021). A list of parameter values used for baseline simulations of the ABM is provided in Table 1 and an algorithm for simulating the ABM is outlined in the Appendix and Figure 9 (adapted from Ghaffarizadeh et al., 2018).

**TABLE 1:** Baseline parameter values used for ABM simulations. For a complete list, see (Norton et al., 2017).

Parameter description	Parameter value	Source
Progenitor cell division limit (*div*_*lim*_)	8–15	See text
Progenitor division probability (*p*_*div*_)	0.05–0.245 per iteration	See text
Stem cell division probability	0.05 per iteration	Norton et al. (2017)
Stem cell symmetric division probability	0.05	Norton et al. (2017)
Initial number of mature vessels	8	Ghaffarizadeh et al. (2018)
Initial number of stem cells	20	Norton et al. (2017)
Initial number of progenitor cells	80	Norton et al. (2017)
High migration rate	8.3 *μ*m per hour	Norton et al. (2017)
Low migration rate	0.83 *μ*m per hour	Norton et al. (2017)
Probability of daughter cell to be a high migratory cell	5%	Norton et al. (2017)
Maximum vessel branching probability	0.2 per iteration	Norton and Popel (2016)
Senescent cell death probability	0.1 per iteration	Norton et al. (2017)

##### 2.2.2.1 Tumor module

The tumor module resides on a 50 × 50 × 50 lattice, in which each cancer cell can only occupy one lattice point. The cancer cells have two proliferative phenotypes: stem cells and progenitor cells, and two migratory phenotypes: high and low migration. Proliferating stem cells have a certain probability of division that remains fixed at predetermined values throughout our simulations. Cancer stem cells are also assumed to have limitless replicative potential (Hanahan and Weinberg, 2000). Progenitor cell behavior is determined by two main input parameters: *p*_div_, the division probability of the cell; and *div*_lim_, the number of times a cell can divide before becoming senescent. A stem cell proliferates less often than a progenitor cell and can divide symmetrically into two stem cells or asymmetrically into a stem cell and a progenitor cell. Progenitor cells can only divide symmetrically into two progenitor cells. Each daughter cell, whether stem or progenitor, has a certain fixed probability of being a high migratory cell.

##### 2.2.2.2 Vasculature module

The vasculature module resides on a 500 × 500 × 500 lattice, which is 10-fold finer than the tumor module lattice because microvessel diameter is typically smaller than the size of a tumor cell (Tsuji et al., 2002; Hao et al., 2018). The initial vasculature is made up of mature segments which are oxygenated. In each simulation step, a new branch or sprout can form at a random location along a mature segment with a certain probability, if there is a hypoxic tumor cell within a certain distance of the vessel. The sprout’s movement is dictated by a tip cell and its length, by proliferating stalk cells. Tip cells migrate towards the nearest source of vascular endothelial growth factor (VEGF) (Gerhardt et al., 2003), which in our model are breast cancer cells (Linderholm et al., 2009). A sprout can fuse with another sprout if the two tips cells are close to one another, or with a stalk cell if the tip cell is close enough to it, through a process called anastomosis. Blood can only flow in new vasculature when such loops are completed (Chaplain et al., 2006). Blood-bearing vessels release oxygen and thus govern normoxic and hypoxic regions within the tumor. Cancer cells in hypoxic regions have a reduced proliferation probability and an increased migration rate (Lin et al., 2012).

##### 2.2.2.3 ABM parameters of interest

Although the ABM has a number of input parameters, those governing progenitor cell proliferation emerge as a natural choice for several reasons. The experimental data comprises tumor volume time-courses, and the bulk of a growing tumor is due to non-stem cancer cells (Morton et al., 2011). Further, since this is a proof of concept study, we wanted to minimize the degrees of freedom, and emphasize ease of visualization. We therefore select *p*_*div*_ and *div*_*lim*_, defined earlier, as ABM parameters of interest.

##### 2.2.2.4 ABM output

From each ABM simulation run at specific values of *p*_div_ and *div*_lim_, we record the number of cancer cells, the number of hypoxic cells, the number of stem cells, and the number of cell divisions, at each iteration. We also collect additional information at the final iteration of the simulation including the locations of all cancer cells and the location of the vasculature within their respective grids. Values of ABM parameters at which we generate output are all possible pairwise combinations from *p*_*div*_ = {0.05, 0.125, 0.245} and *div*_*lim*_ = {8, 12, 15}.

#### 2.2.3 Model selection

We consider three classical models of tumor growth as candidates for our SM, namely, generalized Gompertz, generalized logistic, and Von Bertalanffy. The equations for each of these models can be found in Table 2. To assess goodness of fit and parsimony for each of the models we use AIC (Akaike Information Criterion) and BIC (Bayesian Information Criterion) (Burnham and Anderson, 2004). These are statistical techniques that involve a scoring method that uses the maximum of a log-likelihood function or the residual sum of squares (RSS) to choose the best among candidate models.

**TABLE 2:** Information Criteria (AIC and BIC) for candidate surrogate models. Exp refers to experimental data.

Model	Equation	ABM		Exp	
		AIC	BIC	AIC	BIC
Generalized Gompertz	$\frac{dN}{dt}=N^{\lambda }\left(\delta -\gamma \ln N\right)$	45,696	61,629	5483.8	6793.1
Generalized Logistic	$\frac{dN}{dt}=\gamma N\left(1-\frac{N^{\lambda }}{K}\right)$	43,839	59,771	5483.3	6792.6
von Bertalanffy	$\frac{dN}{dt}=\alpha N^{\gamma }-\beta N$	41,947	57,880	5483.7	6793.0

#### 2.2.4 SM parameter surface reconstruction

For every sampled combination of ABM parameters *p*_div_ and *div*_lim_, we fit the SM model parameters to ABM output by minimizing the weighted sum of squared residuals:

$$\chi^{2}\left({\vec{p}}_{\text{SM}}\right)=\underset{i}{\sum }{\left(\frac{z_{i}-y_{i}\left({\vec{p}}_{\text{SM}}\right)}{\sigma_{i}}\right)}^{2},$$ (2)

where: *z*_*i*_ denotes averaged ABM output generated at time point *i*; *σ*_*i*_, the corresponding standard error; and $y_{i}\left({\vec{p}}_{\text{SM}}\right)$ , the SM output at time point *i* as predicted by parameters ${\vec{p}}_{\text{SM}}$ . We then use the profile likelihood method outlined in (Eisenberg and Jain, 2017), which exploits uncertainty in data (here, ABM output) to infer information on estimated parameters. Specifically, each estimated SM parameter *p*_SM,*i*_ is “profiled” by fixing it across a range of values and the remaining parameters are estimated for each fixed value of *p*_SM,*i*_ (Venzon and Moolgavkar, 1988; Murphy and Van der Vaart, 2000). The maximum value of the likelihood function for each parameter value yields the likelihood profile for that parameter (Eisenberg and Jain, 2017). The likelihood profiles are also used to calculate confidence bounds based on a likelihood threshold. The parameter values at which the profile crosses the threshold (on either side of the optimal parameter value) define the confidence interval at a particular level of significance (Eisenberg and Jain, 2017), here taken to be 95%. Bilinear interpolation—followed by a coordinate transformation for ease of visualization—is used to infer upper and lower bounding hypersurfaces as functions of ABM parameters, for each SM parameter *p*_SM,*i*_.

#### 2.2.5 Estimate SM parameters from experimental data

We now fit the SM model parameters to the xenograft time-course data in (Zhou et al., 2019) by once again minimizing a weighted sum of squared residuals as described in the previous step. Next, we repeat the profile likelihood method described above, but now, with the experimental data. We additionally uncover practically identifiable combinations of SM input parameters, following the approach outlined in (Eisenberg and Jain, 2017). This is done by fitting rational functions (for instance) to the parameter relationships inferred from the profile likelihood graphs (Eisenberg and Hayashi, 2014).

#### 2.2.6 Infer regions of ABM parameters space that correspond to experimental data

Lastly, the identifiable ranges for the data-derived SM parameters found in Step 5 are overlaid on the interpolated map between SM and ABM parameters generated in Step 4 giving us the specific regions of ABM parameter space that correlate with the experimental data. Specifically, for each of our chosen SM parameters, we generate regions in the *p*_*div*_−*div*_*lim*_ (ABM) parameter-plane. The intersection of these regions yield ranges for *p*_*div*_ and *div*_*lim*_ that correspond to a specific choice of our SM parameters. We repeat this process for multiple choices of our SM parameters and take the union of the resulting ABM regions to arrive at the desired ranges for *p*_*div*_ and *div*_*lim*_ that match with the experimental data.

#### 2.2.7 Applying knowledge gained from SMoRe ParS to compare inferred tumor characteristics

Two distinct sets of ABM parameters are chosen from the experimental data-informed region computed in the previous step, namely, *p*_*div*_ = 0.18, *div*_*lim*_ = 9 and *p*_*div*_ = 0.24, *div*_*lim*_ = 11. At each parameter combination, ABM simulations are repeated six times, and used to calculate several metrics to distinguish between the resulting virtual tumors: 1) The Euclidean distance of the farthest cancer cell from the tumor at initiation; 2) the fractal dimension of the tumor vasculature [using MATLAB Central File Exchange file boxcount from F. Moisy (Moisy, 2008)]; 3) the surface area to volume ratio of the tumor; and 4) the compactness of the tumor [using the formula $Comp={\left(Vol\right)}^{1/3}*{\left(36\pi \right)}^{1/6}/\sqrt{SA}$ from (Limkin et al., 2019)]. Here, *SA* refers to the surface area of the tumor and *Vol* refers to the volume of the tumor, calculated as follows. We use the Matlab function alphaShape to find the volume and surface area that encloses all points at which tumor cells are located in the 3D simulation domain. To eliminate confounding effects from tumor cells that have migrated away from the primary tumor mass, any regions of tumor cells smaller than a cutoff threshold of pixel volume = 50 are first removed using the Matlab function RegionThreshold.

## 3 Results

### 3.1 ABM simulations

**FIGURE 3:**
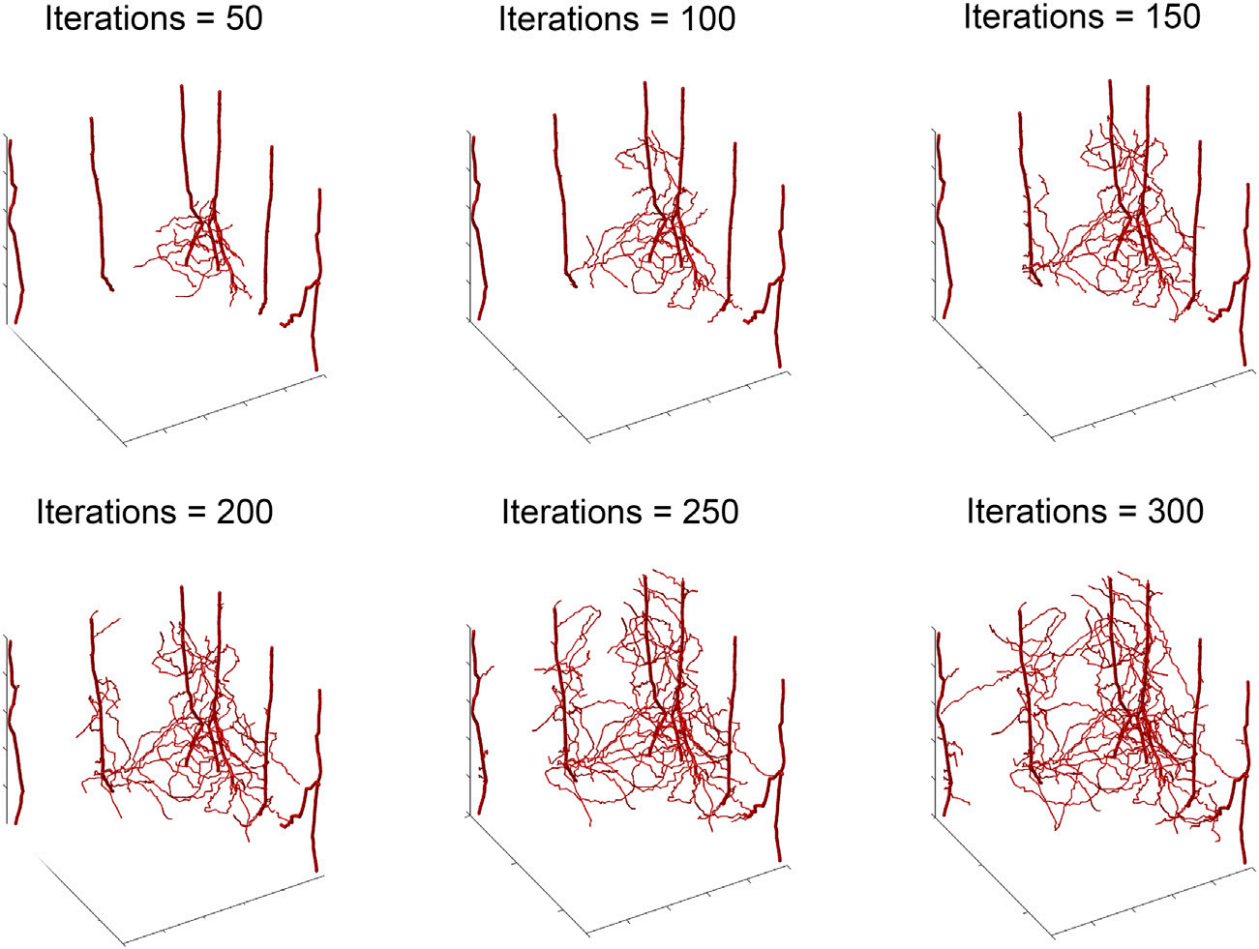
Storyboard showing vasculature evolution within the tumor shown in Figure 2.

To illustrate our ABM of 3D vascular tumor growth, we select representative values of *p*_*div*_ and *div*_*lim*_ at which we generate ABM output. Figure 2 depicts the progression over time of the resultant tumor, showing normoxic (cyan) and hypoxic (purple) tumor cells. Starting from a few cells in the corner of the grid, the tumor expands within the simulation domain as cells proliferate and tumor vasculature evolves. Figure 3 shows the concomitant evolution of tumor vasculature.

### 3.2 Surrogate model selection

The candidate surrogate models are shown in Table 2 along with their information criterion (AIC/BIC) values associated with both ABM output and experimental data. These results show that experimental data alone may not distinguish between the models. However, when fitting to ABM output, the generalized Gompertz (GG) and Logisitic (GL) equations are *e* ^−1875^ and *e* ^−942^ times as probable as the von Bertalanffy (vB) model to minimize information loss, respectively. This means that GG and GL cannot explain the ABM data better than vB. Our results confirm that comparatively the vB growth model provides a better fit to the ABM data. Therefore we select the vB model as our surrogate. This agrees with findings in (Ghaffari Laleh et al., 2022) where these and other test models were fit to tumor volume time-courses from five different data sets.

The vB model has three input parameters (*α*, *β*, *γ*) of which *α* is related to the environmental carrying capacity. This differs significantly between the ABM (1 mm^3^) and the experimental system (∼2,000 mm^3^). Since the two carrying capacities are uncorrelated, *α* cannot function as an interlocutor between the ABM and the experimental data. Therefore, we select *β* and *γ* as our SM parameters of interest.

### 3.3 Parameter surface reconstruction

**FIGURE 4:**
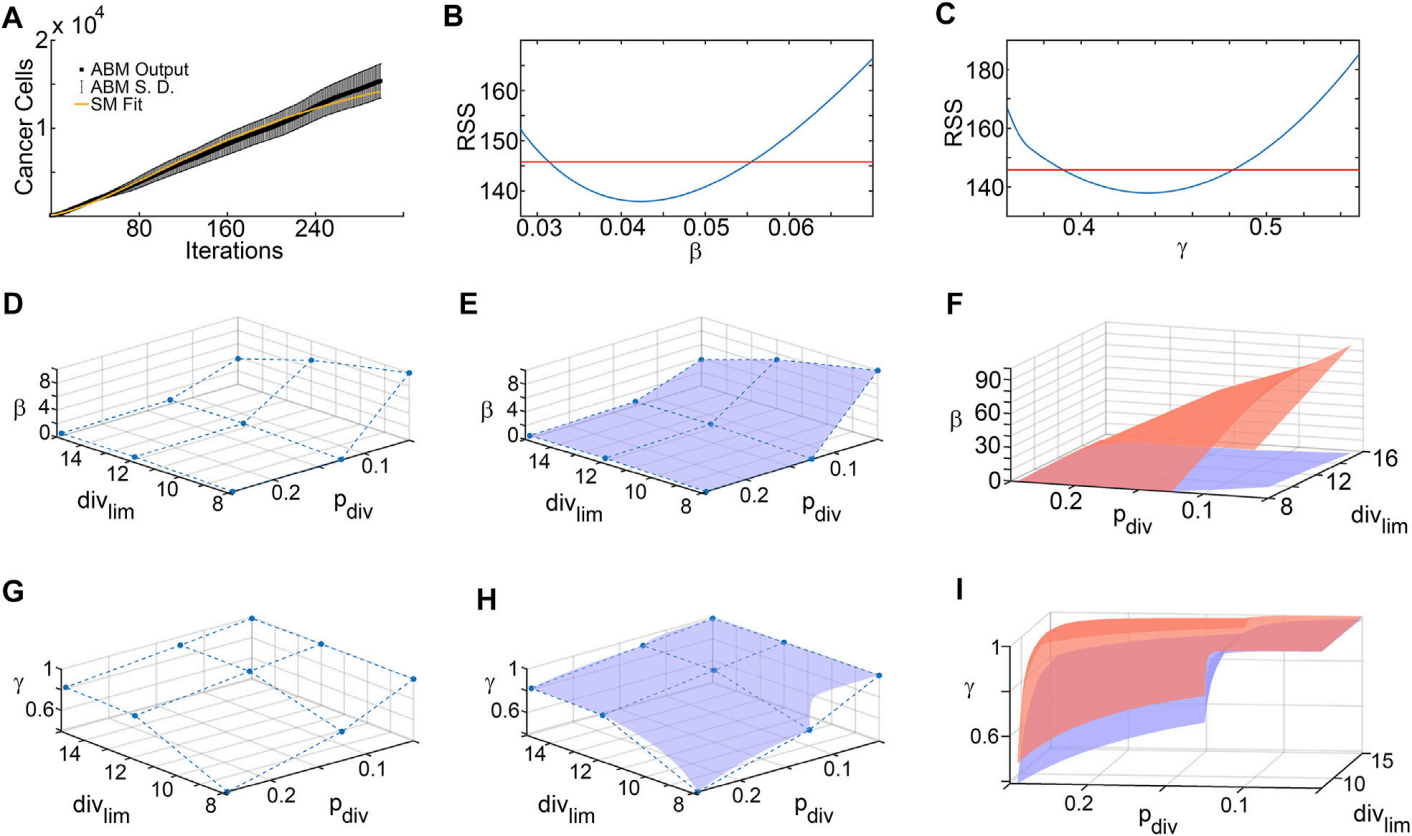
Surrogate model parameter surface reconstruction from ABM output. **(A)** Best fit of surrogate model to averaged ABM output generated with *p*_*div*_ = 0.245 and *div*_lim_ = 8. **(B,C)** Profile likelihoods (solid blue lines) for estimated surrogate model parameters: **(B)** *β*, and **(C)** *γ*. Thresholds for the 95% confidence intervals are shown as red lines and RSS stands for residual sum of squares. The left and right points of intersection of the blue and red curves give the lower and upper bounds, respectively, for the estimated surrogate model parameter, corresponding to these specific values of ABM parameters (*p*_*div*_ = 0.245 and *div*_*lim*_ = 8). **(D–F)** Lower and upper surface reconstruction for *β*. **(D)** Lower bounds for *β* obtained from 95% confidence thresholds like those shown in panel **(B)**, for various choice of ABM parameters *p*_*div*_ and *div*_*lim*_. **(E)** Lower bound surface for *β* reconstructed from the discrete points shown in panel **(D)**. **(F)** Final lower (blue) and similarly reconstructed upper (orange) surfaces for *β*. **(G–I)** Lower and upper surface reconstruction for *γ*, following similar steps.

Figure 4 shows the results of the SM parameter surface reconstruction from ABM output. Figure 4A depicts the best fit of SM output (time-course of # of tumor cells) to ABM output (time-course of # of tumor cells integrated over space) for one specific combination of *p*_*div*_ and *div*_*lim*_. The results of the profile likelihood analysis, quantifying uncertainty in SM parameters *β* and *γ* for this choice of *p*_*div*_ and *div*_*lim*_, are shown in Figures 4B,C. Both parameters are identifiable from the ABM output, as evidenced by u-shaped profiles. The 95% confidence bounds for these fits correspond to discrete points on the upper and lower 95% confidence hypersurfaces in (*p*_*div*_, *div*_*lim*_, *β*) and (*p*_*div*_, *div*_*lim*_, *γ*) space. Repeating this for all sampled combinations of *p*_*div*_ and *div*_*lim*_ yields the sets of discrete points that lie on the upper and lower hypersurfaces of each SM parameter. As an illustration, Figures 4D,G show the discrete points on the lower hypersurfaces for *β* and *γ*, respectively. Next, the surfaces are “filled in” using interpolation, as shown in Figures 4E,H. Finally, Figures 4F,I show the fully reconstructed upper (orange) and lower (blue) hypersurfaces for *β* and *γ*, respectively. For this region of ABM parameter space, we are 95% confident that the SM parameters lie in between these hypersurfaces.

### 3.4 SM parameter estimation from experimental data

**FIGURE 5:**
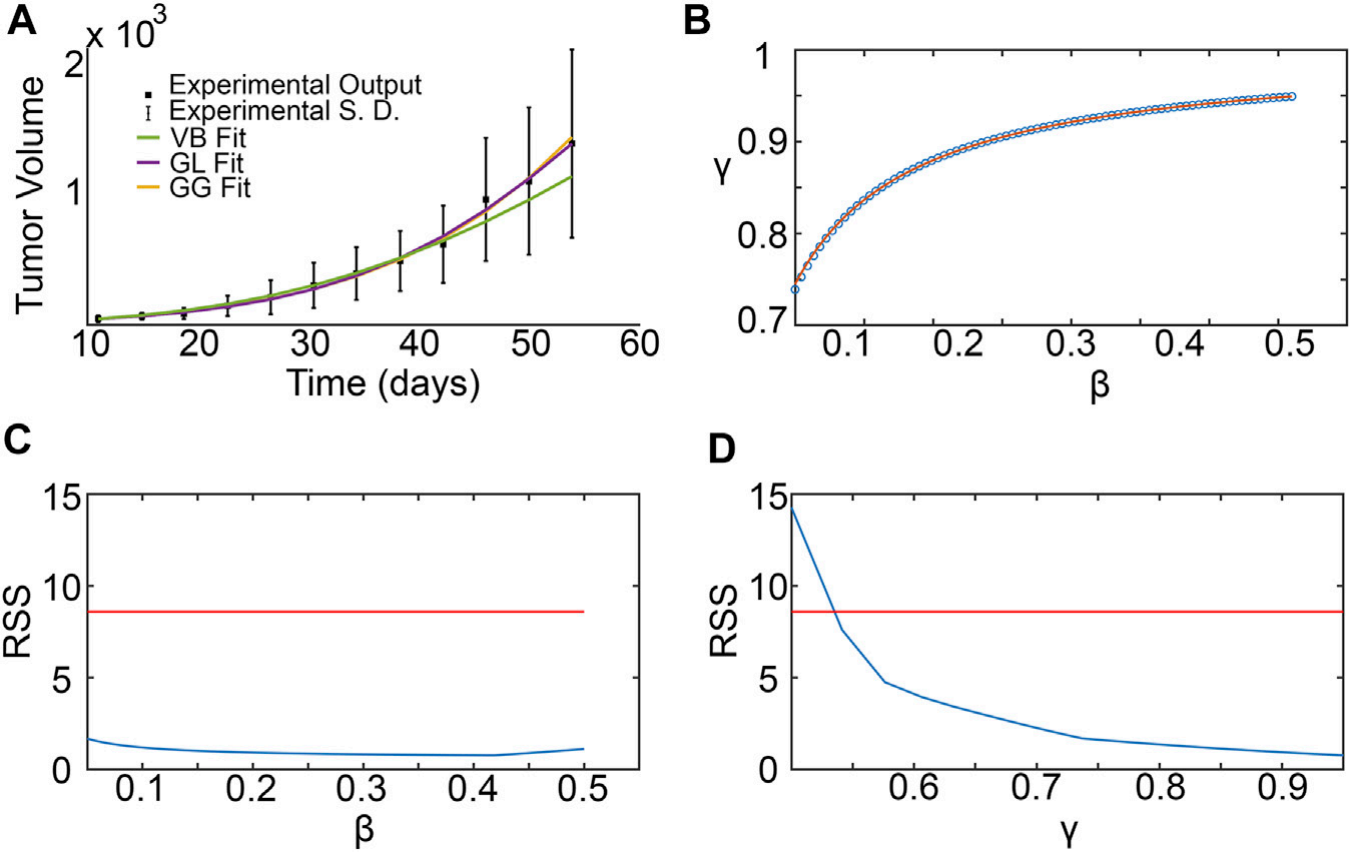
Surrogate model parameter estimation and practical identifiability analysis using breast cancer xenograft data from (Zhou et al., 2019). **(A)** Surrogate model fit to experimental tumor volume time-courses. **(B)** Inferred relationship between *γ* and *β* using the profile-likelihood method (Eisenberg and Jain, 2017), with combinations plotted as blue squares, and potential combination form plotted as a red curve. **(C,D)** Profile likelihoods (solid blue lines) for estimated surrogate model parameters: **(C)** *β*, and **(D)** *γ*. Thresholds for the 95% confidence intervals are shown as red lines and RSS stands for residual sum of squares.

Figure 5A shows the results from fitting the SM parameters *β* and *γ* to the experimental data for breast cancer xenografts taken from (Zhou et al., 2019). From the subset profiles for each parameter graphed in Figures 5C,D, we see that both parameters are practically unidentifiable (or inestimatable) from the experimental data set. Although each parameter on its own is not estimable, the following practically identifiable combination is inferred from parameter relationships between *β* and *γ*, shown in Figure 5B:

$$\gamma \;=\;\frac{\beta +0.0164}{\beta +0.0392}.$$ (3)

We remark that the values for *β* and *γ* depicted in Figure 5B are from within their respective 95% confidence bounds inferred from Figures 5C,D.

### 3.5 Inferring regions of ABM parameter space that correspond to experimental data

**FIGURE 6:**
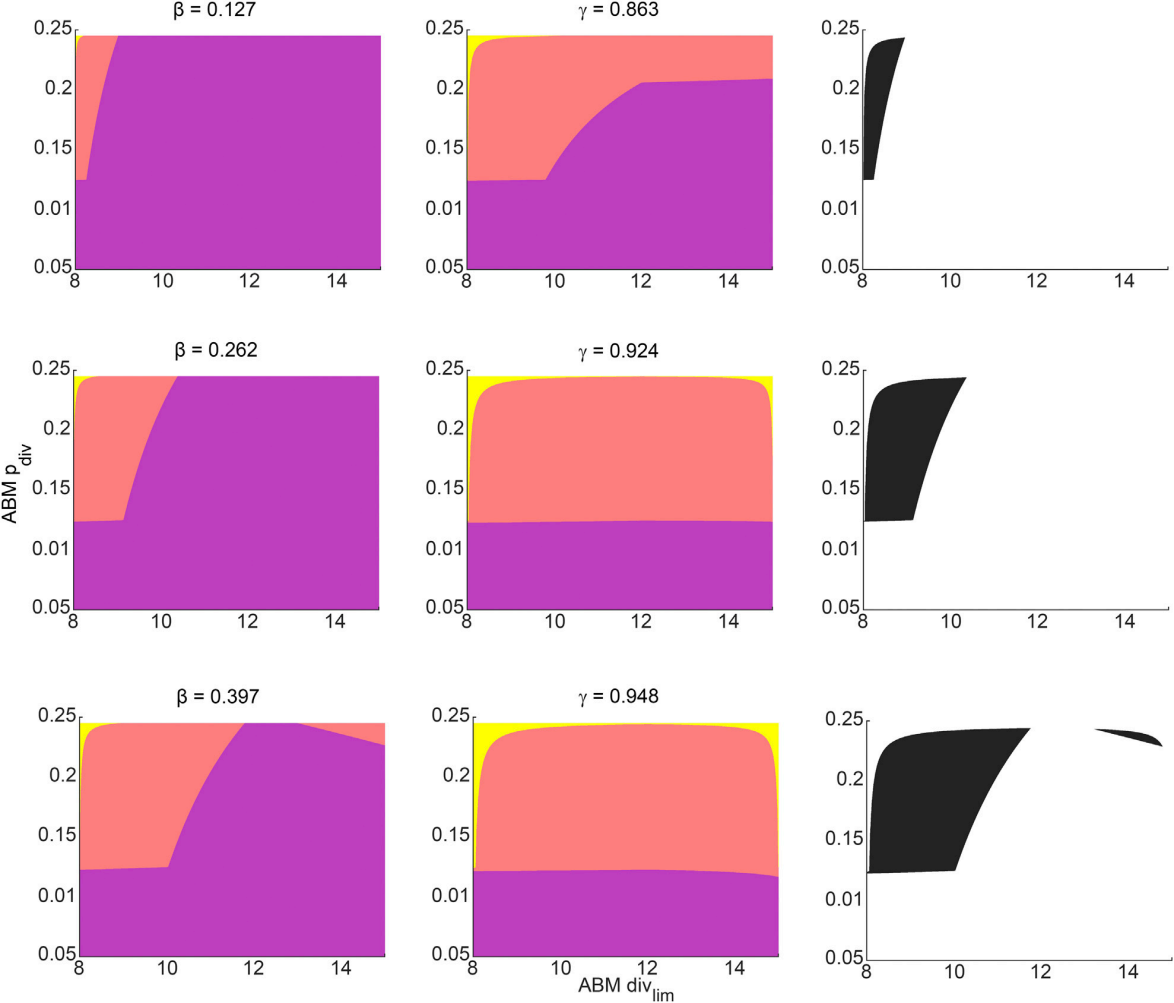
Regions in ABM parameter space corresponding to various choices of surrogate model parameter combinations that were inferred by fitting to experimental data. Orange tinted areas represent regions in ABM parameter space for which that surrogate model parameter lies between its upper and lower reconstructed surfaces. Purple and yellow tinted areas represent (inadmissible) regions when the surrogate model parameter is outside these bounds. The first and second columns represent ABM regions corresponding to various choices of *β* and *γ*, respectively. The third column graphs the intersection of the admissible ABM parameter regions, with each entry corresponding to the pair of *β*-*γ* values from that row. The *β*-*γ* pairs in each row are points that lie on the practically identifiable combination form plotted in Figure 5B.

**FIGURE 7:**
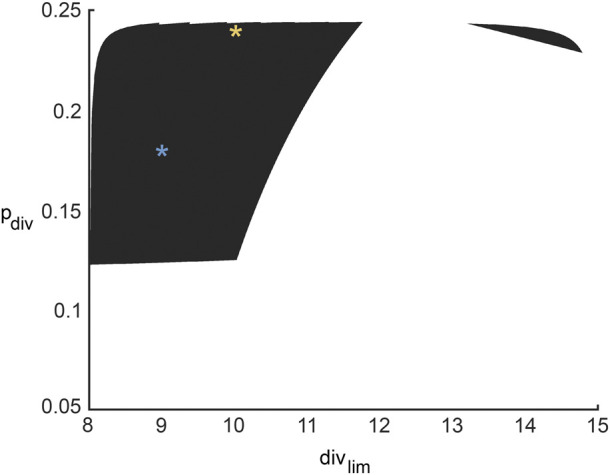
Region in ABM parameter space inferred from all possible surrogate model parameter combinations that fit experimental data equally well. The yellow and blue stars denote parameter sets 1 and 2 (*p*_*div*_ = 0.18, *div*_*lim*_ = 9 and *p*_*div*_ = 0.24, *div*_*lim*_ = 11; respectively, as discussed in Section 3.6 and Figure 8.

For any pair of admissible values of *β* and *γ* as determined by Eq. 3, a corresponding region of ABM parameter space is inferred from Figure 4F for *β*, and Figure 4I for *γ*. The intersection of these two regions gives the region of ABM parameter space that maps to the experimental data for this specific *β*-*γ* combination. Figure 6 shows these inferred regions for three representative pairs of values of *β* and *γ*. Along each row, the first panel shows the ABM parameter region corresponding to that value of *β*, the second panel shows the ABM parameter region corresponding to that value of *γ*, and the third panel shows the intersection of these two regions. Finally, Figure 7 shows the union of several such common regions, for the range of possible values of *β* and *γ*. This is the desired region of ABM parameter space inferred from the experimental data.

### 3.6 Comparing metrics from the ABM parameter space

**FIGURE 8:**
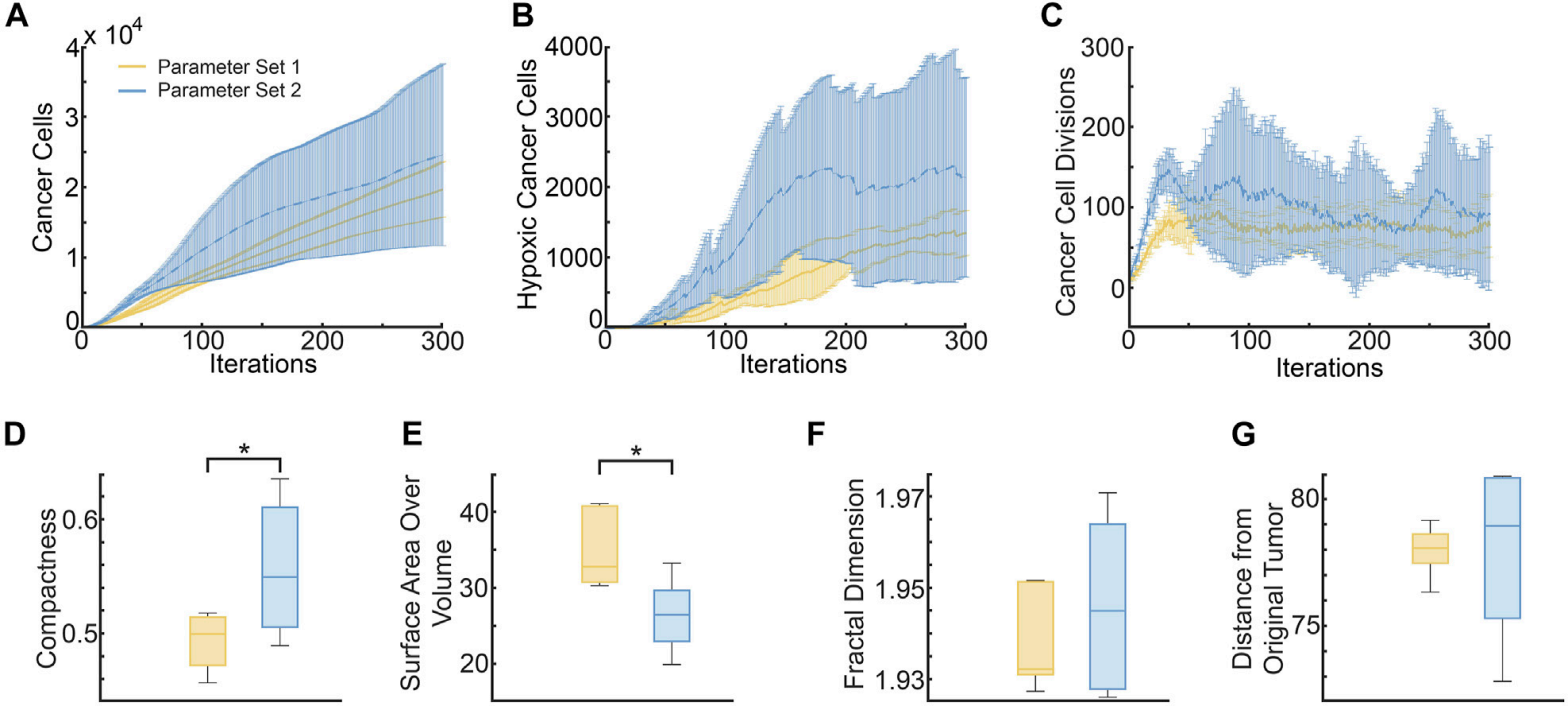
Metrics distinguishing ABM-simulated tumors using parameter set 1 (*p*_*div*_ = 0.18 and *div*_*lim*_ = 9, yellow curves and bars) and parameter set 2 (*p*_*div*_ = 0.24 and *div*_*lim*_ = 11, blue curves and bars). **(A)** Mean and standard deviation of total cancer numbers at each iteration. **(B)** Mean and standard deviation of total number of hypoxic cells at each iteration. **(C)** Mean and standard deviation of total number of cancer cell divisions at each iteration. **(D–G)** Metrics calculated at simulation end-point (iterations = 300), with statistically significant differences indicated. **(D)** Compactness of the simulated tumors. **(E)** Surface area-to-Volume ratio of the simulated tumors. **(F)** Fractal dimension of tumor vasculature. **(G)** Distance of the farthest cancer cell from the origin (location of tumor cells at iteration 0).

**FIGURE 9:**
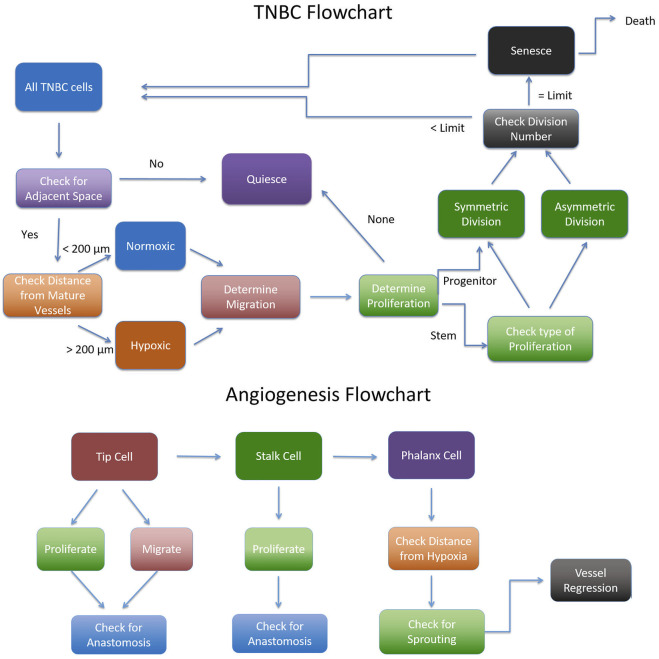
Flowchart for implementing the Agent Based Model adapted from (Norton, 2018).

Two distinct sets of parameters from within the inferred ABM parameter space are chosen to illustrate how the same xenograft volume time-course may come from tumors with very different spatial microstructure. For each parameter set, ABM simulations are repeated six times, and the number of tumor cells, the number of hypoxic cells and the number of cancer cell divisions are recorded at each time step. Additionally, we also calculate the compactness of the tumor, the surface area to volume ratio of the tumor, the fractal dimension of the 3D vasculature, and the distance of the farthest cancer cell from the original tumor, at the end of simulations (iterations = 300). Figure 8 shows how these features compare between the two sets of simulations.

As can be seen from Figure 8A, the mean number of cancer cells of parameter set 2 is consistently larger than parameter set 1, with a difference of about a thousand cells. Parameter set 1 has a relatively small variation between runs as compared to parameter space 2. Similarly, Figure 8B shows that the mean number of hypoxic cells is consistently larger for parameter set 2 than 1. On the other hand, although the number of cancer cell divisions is initially higher for parameter set 2, both sets of simulations stabilize at a similar number (Figure 8C). These findings are unsurprising, given that parameter set 2 allows for a higher probability of division as well as number of allowed divisions, than parameter set 1.

Interestingly, tumors generated from parameter set 2 are significantly more compact than those generated from parameter set 1 (*p*-value = .0357 using a two sample *t*-test, see Figure 8D). This makes sense as parameter set 2 has a larger division probability and cells can divide more times than parameter set 1. Therefore, they should generally reproduce more often and longer before they become senescent, creating a larger, more compact tumor. Although we do see that the variance for parameter space 2 is much larger than parameter space 1, meaning that while they tend to be more compact, there are also cases in which they are less compact, similarly to parameter space 1. In contrast, the surface area to volume ratio is significantly lower for tumors from parameter set 1 than parameter set 2, with a *p*-value of .0149 (see Figure 8E). The average fractal dimensions of the final tumor vasculature are similar between the two parameter sets, with values within 1.93 and 1.97 (see Figure 8F). This is in line with experimental results that found vessels from whole tumor xenografts had fractal dimensions between 1.94 and 2.04 (Kim et al., 2012). Finally, the distance from the original tumor of the farthest cell at the end of the simulations, is also similar between the two parameter sets (see Figure 8G).

## 4 Discussion

There is an unmet need to develop new theoretical and computational frameworks that advance current efforts for making critical connections between computationally complex model (CCM) parameters and real-world data, which can be sparse and highly variable. To that end, we developed SMoRe ParS, which is a potentially paradigm-shifting method for parameter surface reconstruction that tackles this problem. SMoRe ParS envisages an explicitly formulated, data-informed, simpler, surrogate model (SM) as an intermediary that is used to quantify the uncertainty in the relationship between CCM inputs and SM parameters, and also between SM parameters and real-world data. SM parameters, thus, serve as a link between difficult-to-estimate CCM inputs and noisy data and enable calibration and uncertainty quantification of CCM parameters that map directly onto an experimental data set.

To illustrate the capability of SMoRe ParS to connect CCM output and real-world data, we applied it to an ABM of 3D vascular tumor growth as the CCM, and data from tumor xenograft growth experiments as real-world data. Our method allowed us to construct an explicit mapping between ABM parameters and tumor volume time-courses, which encodes within it information on uncertainty in inferred parameter values. We then selected two distinct sets of ABM parameters that map onto the same data set, to investigate any differences between the resultant simulated tumors. Indeed, several trends distinguished the two sets of simulations. Variances in tumor cell number time-courses shown in Figure 8 suggest that parameter set 1 (lower probability of cell division and fewer number of allowed cell divisions) is consistent with a slow growing tumor, whereas parameter set 2 (higher probability of cell division and greater number of allowed cell divisions) allows for both slower and faster growing tumors. In fact, the variation within parameter set 1 was consistently smaller than within parameter set 2 across all metrics, except surface area to volume ratio (Figure 8). This suggests that while in parameter set 1 all tumors grew relatively similarly, in parameter set 2 the randomness of which cells could proliferate or move could lead to a substantial difference in the growth rate of the tumor. This is consistent with previous results that showed if cells are surrounded by other cells, even if their proliferation probability is high, they will not be able to divide because there is not enough space, thus limiting the overall growth of the tumor (Norton et al., 2017). Therefore, tumors that have the capability of excessive growth may not be able to do so under certain conditions where their growth is limited by spatial inhibition. This also explains the trends in compactness and surface area to volume ratio of the parameter sets. Tumors generated from parameter set 1 were less compact than those from parameter set 2, with a higher surface area-to-volume ratio, indicating more space to grow. Both these metrics have been suggested as predictors of malignancy in lung and head and neck cancers Aerts et al. (2014), Bogowicz et al. (2017), He et al. (2014), Wang et al. (2016). Our results suggest that tumors with very distinct malignant potential could be “hiding” within aggregate, macroscopic data.

In this paper, we chose to select the SM from a set of phenomenological models because our main concern was providing an easy to follow proof of concept example for cellular-level tumor growth. In other applications, where for example molecular or microenvironmental drivers of tumor progression and treatment response are of interest, it is possible to choose a mechanistic formulation of the surrogate. There are several advantages to doing so, including being able to more fully leverage the SM’s ability to directly connect the ABM to the experimental data. A mechanistic SM would also have stand alone value as it provides a more complete characterization of the system and can be used for long term forecasting with greater confidence. We remark that in our approach, only a handful of ABM parameters can be considered at a time. In general, the precise number would depend on the computational complexity of the ABM and SM and how much experimental data is available. Further, deriving a mechanistic SM that can match both the experimental data and the ABM output may prove time-consuming. While we use cancer as an illustrative example and as the subject of our future studies, SMoRe ParS can easily be applied to a wide range of CCMs for basic biology and translational systems biology investigations.

## Data Availability

The original contributions presented in the study are included in the article/supplementary material, further inquiries can be directed to the corresponding author.

## Appendix A

### Agent based model

The cancer agent-based model is made up of two main modules: the vascular module and the cancer cell module, as described in Section 2.2.2. Figure 9 shows a flow chart of how the ABM is implemented.

### Model setup

The cancer module is initialized with 100 cells, of which 20 are stem cells and 80 are progenitor cells. 5% of all cells are assumed to have a higher migratory speed. Each progenitor cell may divide at most *div*_*lim*_ times before undergoing senescence, and the initial pool of progenitor cells are randomly assigned a division cycle count between 1 and *div*_*lim*_. The vascular module is initialized with eight capillaries that are aligned along the edges of the simulation grid, with two capillaries branching off of another. The floor of the simulation domain is assumed to rest on healthy tissue and acts as a constant source of oxygen.

### Vascular module

The vascular module starts to evolve once hypoxic cancer cells appear in the simulation. These are an assumed source of angiogenic factors such as VEGF. A cancer cell becomes hypoxic once it is 200 microns away from a source of oxygen, namely a mature capillary or the floor of the simulation domain. The vascular network evolves as follows. In each iteration, a cell lining a capillary has a chance to generate a new tip cell, determined by local hypoxic conditions. Each active tip cell determines whether it migrates or proliferates. A tip cell can only proliferate if there is no stalk cell in the sprout, in which case the tip cell proliferates to produce a stalk cell behind it. Tip cells with stalk cells behind them do not proliferate. Once a tip cell has proliferated, we test whether it is adjacent to another tip cell or vascular segment and if so, the two tip cells or the tip cell and vascular segment anastomose. This results in the formation of a closed loop which represents a blood-bearing vessel that is a source of oxygen. All segments in such a vessel are then marked as mature and can no longer proliferate or migrate. If the tip cell does not proliferate, it checks whether it can migrate. We introduce a variable *migdist*, which determines how far the tip cell migrates. *migdist* cannot exceed more than 1.5 times the length of the tip cell, and is calculated based on the local VEGF concentration. This, in turn, is a function of the number of neighboring cancer cells. Details on how *migdist* is computed are provided in (Norton and Popel, 2016). The tip cell randomly migrates towards one of its neighboring cancer cells, excluding cells that would cause the tip cell to migrate backwards. The tip cell does not migrate if it would cause it to leave the vascular grid. After migration, the tip cell checks whether it can anastomose, as described previously.

The second step of the vascular module involves stalk cell decisions. Stalks cells’ main function is to proliferate and push the tip cell forward. A stalk cell only proliferates when it reaches the end of its cell cycle, and if there is enough space. If a stalk cell proliferates, a new stalk cell is created replacing the old tip cell. Afterwards a new tip cell is created of 1 micron length in the direction of the old tip cell. The old stalk cell then becomes a quiescent phalanx cell which cannot proliferate or migrate. The new stalk cell resets its cell cycle and the tip cell checks if it should anastomose.

The last step of the vascular module allows for vessel branching of phalanx cells. Neither tip cells nor stalk cells are allowed to branch. Branching occurs due to the presence of nearby hypoxic cells. Specifically, the phalanx cell can only branch if there are hypoxic cells within 250 microns of it. The new branch creates a tip cell that is extended in the direction of the nearest hypoxic cancer cell. Once a phalanx cell has branched the two cells next to it cannot branch.

### Cancer module

The cancer module runs through each cancer cell in a random order so as not to introduce bias. Each cancer cell can migrate, proliferate, quiesce, senesce and/or die in each iteration. First, the cell determines whether it is normoxic or hypoxic by checking whether it is less than 200 microns from a mature vessel. Hypoxic cells are more migratory, increasing the speed they migrate 3-fold, and are less proliferative, decreasing their chances to proliferate by half. In order for the cancer cell to migrate or proliferate there must be available space. If there is no available space, the cell becomes quiescent. The cell decides whether it will migrate based on its migration probability. The number of voxels the cell migrates is based on its migration speed. Therefore, each migrating cell randomly chooses an open space to migrate into and repeats this as many times as its migration speed.

The next decision the cancer cell makes is whether it can proliferate. Each cancer cell has its own proliferation rate depending on whether it is a stem cell or a progenitor cell and whether it is hypoxic or not. If it is a progenitor cell, it can only proliferate if it has not reached its division limit, *div*_*lim*_. In this case, the progenitor cell decides whether it will divide based on its proliferation probability *p*_*div*_. If it decides to proliferate, the progenitor creates a new progenitor cell in a random adjacent grid space and increases its division number by 1. The new progenitor cell inherits the parent cell’s division number and has a 5% probability of being highly migratory. Once a progenitor cell has reached its division limit, it becomes senescent. Alternatively, if the current cancer cell is a stem cell, it first decides whether it will divide based on its proliferation probability. Then it decides whether it will divide symmetrically into another stem cell or asymmetrically into a progenitor cell. Stem cells have no division limit but if a stem cell creates a new progenitor cell, the new progenitor cell has a full division limit of *div*_*lim*_. At the end of the simulation, any cell that has been hypoxic for 40 iterations dies. Each senescent cell has a 10% probability of dying in each iteration.
